# Supplementary figures and images for: Anticipation of ventricular tachyarrhythmias by a novel mathematical method: Further insights towards an early warning system in implantable cardioverter defibrillators
Source: PLoS One. 2020 Oct 1;15(10):e0235101. doi: 10.1371/journal.pone.0235101 (PMC7529227; doi:10.1371/journal.pone.0235101)

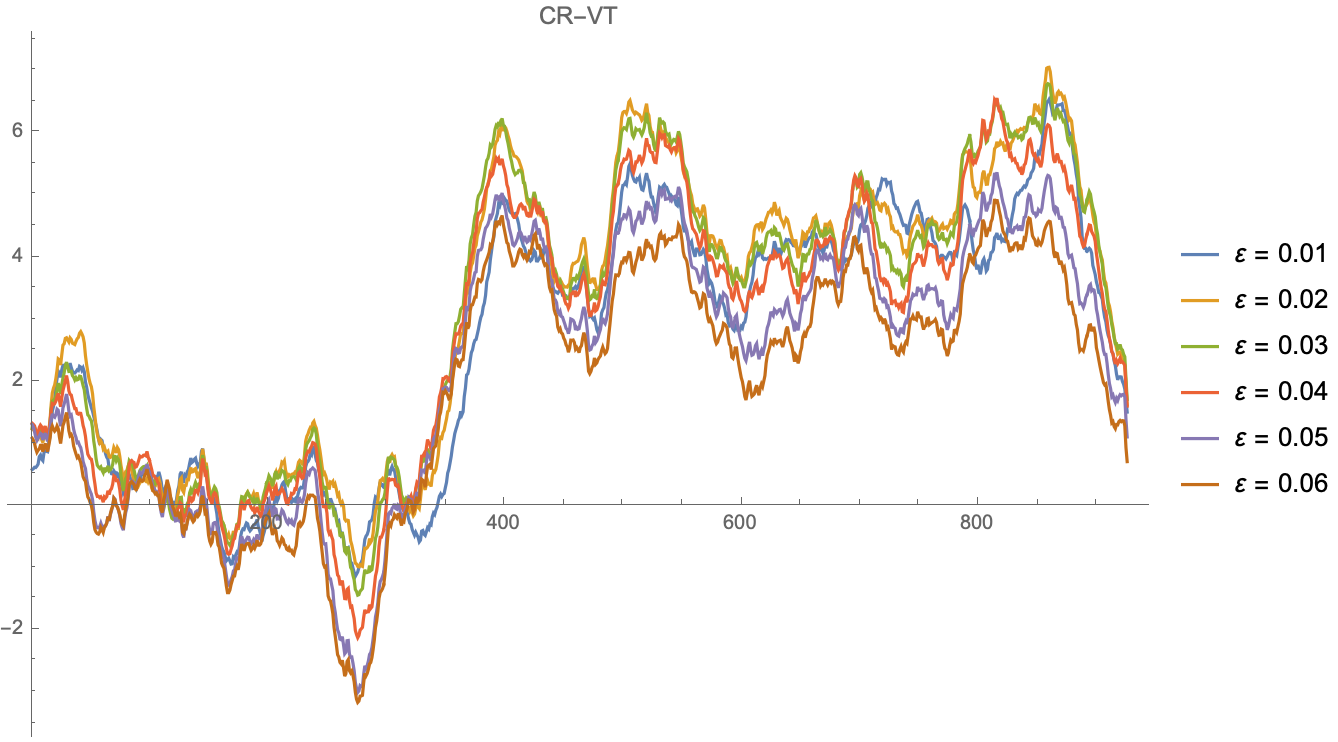

Supplement: S1 Fig — The regular graphs are derived considering ε values of 0.01, 0.02, 0.03, 0.04, 0.05, 0.06 and a window of 60 points. The degree centrality is averaged for each window and plotted in this figure. (TIFF) [file pone.0235101.s002.TIFF]
