# Supplementary material for: Anticipation of ventricular tachyarrhythmias by a novel mathematical method: Further insights towards an early warning system in implantable cardioverter defibrillators
Source: PLoS One. 2020 Oct 1;15(10):e0235101. doi: 10.1371/journal.pone.0235101 (PMC7529227; doi:10.1371/journal.pone.0235101)
Supplement: Attachment — Submitted filename: PLOS ONE REPLY 2020.docx [file pone.0235101.s003.docx]

**August 10^th^, 2020**

**Elena G. Tolkacheva, PhD
Academic Editor
PLOS ONE**

**Journal: PLOS ONE**

**Manuscript ID: PONE-D-20-17194
Title: Anticipation of ventricular tachyarrhythmias by a novel mathematical method: Further insights towards an early warning system in implantable cardioverter defibrillators**

**Authors: Gabriel S. Zamudio, Manlio F. Márquez, Marco V. José**

Dear Dr. Tolkacheva:

Thank you for your email dated July 29^th^, 2020 and for the reviewer’s comments. We are thankful to the reviewers for their helpful criticisms. We carefully evaluated and answered all the questions posed by reviewers. Please check below.

**Answer to reviewers**

Reviewers' comments:

Reviewer's Responses to Questions

**Comments to the Author**

1. Is the manuscript technically sound, and do the data support the conclusions?

The manuscript must describe a technically sound piece of scientific research with data that supports the conclusions. Experiments must have been conducted rigorously, with appropriate controls, replication, and sample sizes. The conclusions must be drawn appropriately based on the data presented.

Reviewer #1: Yes

Reviewer #2: Yes

2. Has the statistical analysis been performed appropriately and rigorously?

Reviewer #1: Yes

Reviewer #2: Yes

3. Have the authors made all data underlying the findings in their manuscript fully available?

The [PLOS Data policy](about:blank#sharing) requires authors to make all data underlying the findings described in their manuscript fully available without restriction, with rare exception (please refer to the Data Availability Statement in the manuscript PDF file). The data should be provided as part of the manuscript or its supporting information, or deposited to a public repository. For example, in addition to summary statistics, the data points behind means, medians and variance measures should be available. If there are restrictions on publicly sharing data—e.g. participant privacy or use of data from a third party—those must be specified.

Reviewer #1: Yes

Reviewer #2: Yes

4. Is the manuscript presented in an intelligible fashion and written in standard English?

PLOS ONE does not copyedit accepted manuscripts, so the language in submitted articles must be clear, correct, and unambiguous. Any typographical or grammatical errors should be corrected at revision, so please note any specific errors here.

Reviewer #1: Yes

Reviewer #2: Yes

5. Review Comments to the Author

Please use the space provided to explain your answers to the questions above. You may also include additional comments for the author, including concerns about dual publication, research ethics, or publication ethics. (Please upload your review as an attachment if it exceeds 20,000 characters)

Reviewer #1: This is an interesting mathematical approach to look at heart rate variability and its ability to predict VT events. The analytical approach is sufficiently novel to be of interest, and it appears to show an ability to discriminate RR interval time series preceding VT events from control RR interval time series from the same patients. However, we are only really shown a representative example of time series analysis and associated derived metrics (Figure 3) and there is no tabulation or graphical representation of this for all 81 patients. So it is not clear that this is genuinely a "representative" result, or the single best time series in which discrimination occurred. Presentation of the results from the whole cohort would address this.

The result of the analysis of the 81 patients can be seen in this plot:

There is a great variability among each individual patient.

In **Discussion** we added the following paragraph:

“The process of optimization of the epsilon parameter value requires a more extensive clinical experimentation. It stands to reason that the parameter value is specific for each individual person and ought to be tuned from the complete set of clinical parameters of the patient to avoid false-positives and false-negatives. In a more general case, it is also probable that the parameter value is not fixed throughout the day and is dependent of the circadian rhythm of the patient.”

The discussion should note that a significant clinical limitation of the work is that this type of approach is unlikely to work in patients with AF. Because the AF group with ICDs is currently a significant challenge to us clinically, this is an important limitation.

Thank you very much for pointing out this important limitation. We have now included a comment in Limitations as follows:

“A significant clinical limitation of this work is the fact that this approach is restricted to patients with normal sinus rhythm and is unlikely to work in patients with atrial fibrillation or those with a pacemaker. In the former group because of the large variability of R-R intervals, and in the later because of fixed pacing rhythms.”

Reviewer #2: In this manuscript, the authors developed a method to transform time series into graphs which is sensitive to a single parameter. And they also demonstrated the feasibility of using this method to detect early warning of VA episode. The followings are my comments to further improve this manuscript.
1. In figure 1, the authors gave an example to using ε-regular graph and compare their method with visibility graph. The author could also show the result of visibility graph in figure 1 to make the comparison more straightforward.

We modified Figure 1 to include Figure 1C that shows the transformation of the time series using the visibility graph algorithm.

2. From figure 2, the authors concluded “The degree distributions for the graphs constructed form the Brownian motion approximates a Gaussian curve despite its multiple modes; and the distribution from points sampled from a normal distribution approximates a lognormal distribution.” The authors simply said what they observed in the plot. They should further explain the results.

We have now included a comment in Methods as follows:

“The distributions of the degree measurements maintain, up to some extent, the statistical properties from the time series they were derived from. The difference between any two values of the Brownian motion follows a normal distribution, and this property is shown by the *epsilon-regular graphs* in the degree centrality measure when setting the epsilon parameter equal to values related to the standard deviation.”

3. The authors show the complete data in supplementary files instead of providing the website source.

## We now provide the website source of the data in the format requested by the journal guidelines, also, the URL of the websites are cited in the section Application to Imminent VT.

4. In figure 3, the authors only showed the graph with ε = 0.04, why is this? They should sweep this parameter and show how the graph looks like with different parameter values. They should also explain how they optimize this parameter.

The parameter epsilon = 0.04 showed the best results for the current application. We have added the experimental results for the parameter values of εpsilon = 0.01, 0.02, 0.03, 0.05, and 0.06 in Supporting Information II. We have added the following paragraph in Application to Imminent VT section:

“The optimization of the parameter εpsilon is based on the statistical parameters of the R-R time series. The average, minimum and maximum distance between two points of the R-R time series are: 0.043, 1.11x${10}^{-16}$, 0.12, respectively. Thus, the εpsilon value of 0.04 approximates the mean of the differences. In Supporting Information II, we show the degree time series when the values 0.01, 0.02, 0.03, 0.04, 0.05, and 0.06 are assigned to the parameter epsilon (Fig S1). Note that the overall pattern is preserved. In particular, the abrupt change of the dynamics is captured by the different values of the parameter epsilon”.

5. “We propose this change in the dynamic of the R-R intervals as a measurable and detectable early warning of the VT event, occurring an average of 514.625 seconds (8:30 minutes) before the start of the VT episode.” How did the authors get this number (514.625) from the graph? They should provide the details.

# In Application to Imminent VT we added the following sentence:

“The count of 514.625 seconds corresponds to the sum of the average of the time lapses of the last R-R intervals starting from the point 276 to when the VT episode begins at point 985.”

6. PLOS authors have the option to publish the peer review history of their article ([what does this mean?](about:blank#loc-peer-review-history)). If published, this will include your full peer review and any attached files.

If you choose “no”, your identity will remain anonymous but your review may still be made public.

**Do you want your identity to be public for this peer review?** For information about this choice, including consent withdrawal, please see our [Privacy Policy](about:blank).

Reviewer #1: No

Reviewer #2: No

 [NOTE: If reviewer comments were submitted as an attachment file, they will be attached to this email and accessible via the submission site. Please log into your account, locate the manuscript record, and check for the action link "View Attachments". If this link does not appear, there are no attachment files.]

While revising your submission, please upload your figure files to the Preflight Analysis and Conversion Engine (PACE) digital diagnostic tool, [https://pacev2.apexcovantage.com/](about:blank). PACE helps ensure that figures meet PLOS requirements. To use PACE, you must first register as a user. Registration is free. Then, login and navigate to the UPLOAD tab, where you will find detailed instructions on how to use the tool. If you encounter any issues or have any questions when using PACE, please email PLOS at [figures@plos.org](about:blank). Please note that Supporting Information files do not need this step.

All in all, we do thank the reviewers for their helpful comments and criticisms; for we feel that they have helped us to improve the quality and presentation of the paper. We expect that the present version of the manuscript answers all their concerns.

Yours Sincerely,

Marco V. José PhD

Theoretical Biology Group

Instituto de Investigaciones Biomédicas

Universidad Nacional Autónoma de México

Apartado Postal 70228 Ciudad Universitaria

C.P. 04510 CDMX, México

Tel: 52-55-5622-3894

Mobile: 52-55-1320-6111

Author for correspondence: Marco V. José ([marcojose@biomedicas.unam.mx](mailto:marcojose@biomedicas.unam.mx))
